# Patterns of attentional bias in antenatal depression: an eye-tracking study

**DOI:** 10.3389/fnbeh.2023.1288616

**Published:** 2023-12-05

**Authors:** Yao Xu, Peiwen Zheng, Wenqian Feng, Lipeng Chen, Shiyu Sun, Jie Liu, Weina Tang, Ciqing Bao, Ling Xu, Dongwu Xu, Ke Zhao

**Affiliations:** ^1^School of Mental Health, Wenzhou Medical University, Wenzhou, China; ^2^Sichuan Provincial Center for Mental Health, Sichuan Academy of Medical Science and Sichuan Provincial People's Hospital, Chengdu, China; ^3^Shaoxing 7th People’s Hospital, Shaoxing, China; ^4^Wenzhou Seventh People’s Hospital, Wenzhou, China; ^5^Lishui Second People’s Hospital Affiliated to Wenzhou Medical University, Lishui, China; ^6^The Affiliated Kangning Hospital of Wenzhou Medical University Zhejiang Provincial Clinical Research Center for Mental Disorder, Wenzhou, China

**Keywords:** antenatal depression, attentional bias, eye tracking, pregnancy, emotional pictures

## Abstract

**Introduction:**

One of the most common mental disorders in the perinatal period is depression, which is associated with impaired emotional functioning due to alterations in different cognitive aspects including thought and facial emotion recognition. These functional impairment may affect emerging maternal sensitivity and have lasting consequences for the dyadic relationship. The current study aimed to investigate the impact of depressive symptoms on the attention bias of infant stimuli during pregnancy.

**Methods:**

Eighty-six pregnant women completed the Edinburgh Postnatal Depression Scale and an eye-tracking task comprising infant-related emotion images. All participants showed biased attention to infant-related images.

**Results:**

First, compared to healthy pregnant women, pregnant women with depression symptoms initially directed their attention to infant-related stimuli more quickly (*F* (1, 84) = 6.175, *p* = 0.015, *η^2^* = 0.068). Second, the two groups of pregnant women paid attention to the positive infant stimuli faster than the neutral infant stimuli, and the first fixation latency bias score was significantly smaller than that of the infant-related negative stimulus (*p* = 0.007). Third, compared with the neutral stimulus, the non-depression group showed a longer first gaze duration to the negative stimulus of infants (*p* = 0.019), while the depressive symptoms group did not show this difference.

**Conclusion:**

We speculate that structural and functional changes in affective motivation and cognitive-attention brain areas may induce these attentional bias patterns. These results provide suggestions for the implementation of clinical intervention programs to correct the attention bias of antenatal depressed women.

## 1 Introduction

Antenatal depression has received increasing attention in recent years. According to recent estimates, the prevalence of antenatal depression in high-income countries ranges from 7 to 20% (Andersson et al., 2003; Melville et al., 2010), and in low and middle-income countries, the prevalence is greater than 20% (Gavin et al., 2011; Husain et al., 2012). Antenatal depression is mainly manifested as an inability to concentrate, anxiety, extreme irritability, poor sleep, easily fatigued or a persistent sense of fatigue, constantly wanting to eat or no appetite, not interested in anything, inability to pick up one’s spirit, persistent depression, wanting to cry, and unstable moods (Sandman et al., 2012; Glover, 2014). Compared with general depression, antenatal depression affects not only the patient but also the offspring and even the entire family. Antenatal depression is associated with adverse fetal and maternal outcomes, including fetal malformations, low birth weight, preterm delivery, stillbirth, fetal distress, and obstetric complications such as placental abruption and postpartum hemorrhage (Alder et al., 2007; Bansil et al., 2010; Staneva et al., 2015; Wallwiener et al., 2019); it also has an ongoing effect on the growth and development of the offspring, either directly or indirectly. The offspring of women with antenatal depression are at higher risk of behavioral/emotional problems such as attention deficit hyperactivity disorder (ADHD), childhood autism (O’Connor et al., 2002; Walder et al., 2014), depression, impulsivity, pubertal cognitive impairment (Van den Bergh et al., 2005; Pawlby et al., 2009), and schizophrenia in adulthood (Maki et al., 2010).

A large number of studies have shown that many factors contribute to the occurrence of depression. Cognitive factors, especially cognitive bias (bias in processing negative stimuli), are important contributors to the occurrence, persistence, and development of depressive symptoms (Taylor and John, 2004). Antenatal depression is a subtype of depression in which cognitive processes are equally important to the development of symptoms (Roomruangwong et al., 2016). Attention is the first step in the cognitive process and also the main means for individuals to deal with external stimuli (Treisman, 1964; Rueda et al., 2023). Attention also affects higher brain functions such as language, learning, computation, and emotion (D'Souza et al., 2020). We speculate that attentional bias may be the initial stage of the negative cognitive bias characteristic of depression.

However, most previous studies of attention have been based on the measurement of response time, which can only test the attentional performance of an individual at a single time point; such an approach cannot continuously test the entire attentional process of an individual over a certain period (Chen et al., 2012). Therefore, when evaluating attentional bias test results based on response time, it is difficult to distinguish early attentional engagement with the stimulus from subsequent attentional shift (Clarke et al., 2013). Compared to reaction time tasks, the eye-tracking technique offers a more direct and continuous measurement of visual attention. It effectively captures changes in attention throughout a task and provides intuitive evidence and predictions regarding the attentional state (Ahonniska-Assa et al., 2018).

Human and non-human animal studies have demonstrated that substantial structural and functional changes occur in the brain during pregnancy and postpartum (Brunton and Russell, 2008; Barba-Muller et al., 2019). These changes may have an effect on attentional function. Studies have shown that the volume of gray matter in the brains of pregnant women is decreased, including the hypothalamus. The hypothalamus is an area of the midbrain area at the edge of the cortical neural circuits, which is associated with mother’s response to her baby, and also plays a role in attentional function (Hoekzema et al., 2017). Meanwhile, research indicates that pregnant and postpartum women exhibit deficits in working memory performance and information processing compared to non-pregnant women (Henry and Rendell, 2007). These evidences suggested that women may have changes in brain structure and function during pregnancy, which might be to adapt to the physiological changes of pregnancy and better care for offspring.

At present, there is a scarcity of research investigating whether changes in brain structure and function affect the attention bias of pregnant women. Previous studies have found that healthy pregnant women tend to have an attentional bias toward emotion-related images of their infants (Lucion et al., 2017; Li et al., 2019). This attention bias helps mothers to take care of their children. One of our previous eye movement studies found that women with perinatal depression had a significant bias toward negative stimuli (Tang et al., 2019). However, this study only utilized general mood pictures and did not explore the patterns of attention toward infant-related stimuli in women with antenatal depression. Therefore, in this study, we further explored whether pregnant women have an attentional bias toward emotional images associated with infants, and whether this attentional bias is altered in pregnant women with depressive symptoms.

In summary, the following hypotheses were proposed: (1) pregnant women will have an attentional bias toward infant-related emotional images compared to neutral images. (2) The attention pattern of women with antenatal depressive symptoms will be different from that of healthy pregnant women.

## 2 Methods

### 2.1 Participants

A total of 86 pregnant women took part in this study. The participants were recruited from the obstetric outpatient clinic of the First Affiliated Hospital of Wenzhou Medical University from December 2018 to April 2019. All participants in this study provided written informed consent and voluntarily agreed to participate. This study was approved by the Ethics Committee of Wenzhou Medical University.

The inclusion criteria for this study were as follows: (1) right-handed; (2) between the ages of 18 and 40 years; (3) normal color vision with the naked eye or corrected vision; (4) voluntarily provided written informed consent. The exclusion criteria were: (1) serious pregnancy-related complications (preeclampsia, intrauterine growth restriction, or gestational diabetes); (2) serious medical or neurological conditions, and no substance dependence (except caffeine) in the past year; and (3) history of severe psychiatric conditions (e.g., psychotic or bipolar disorders) according to the fifth edition of the Diagnostic and Statistical Manual of Mental Disorders.

**Figure 1:**
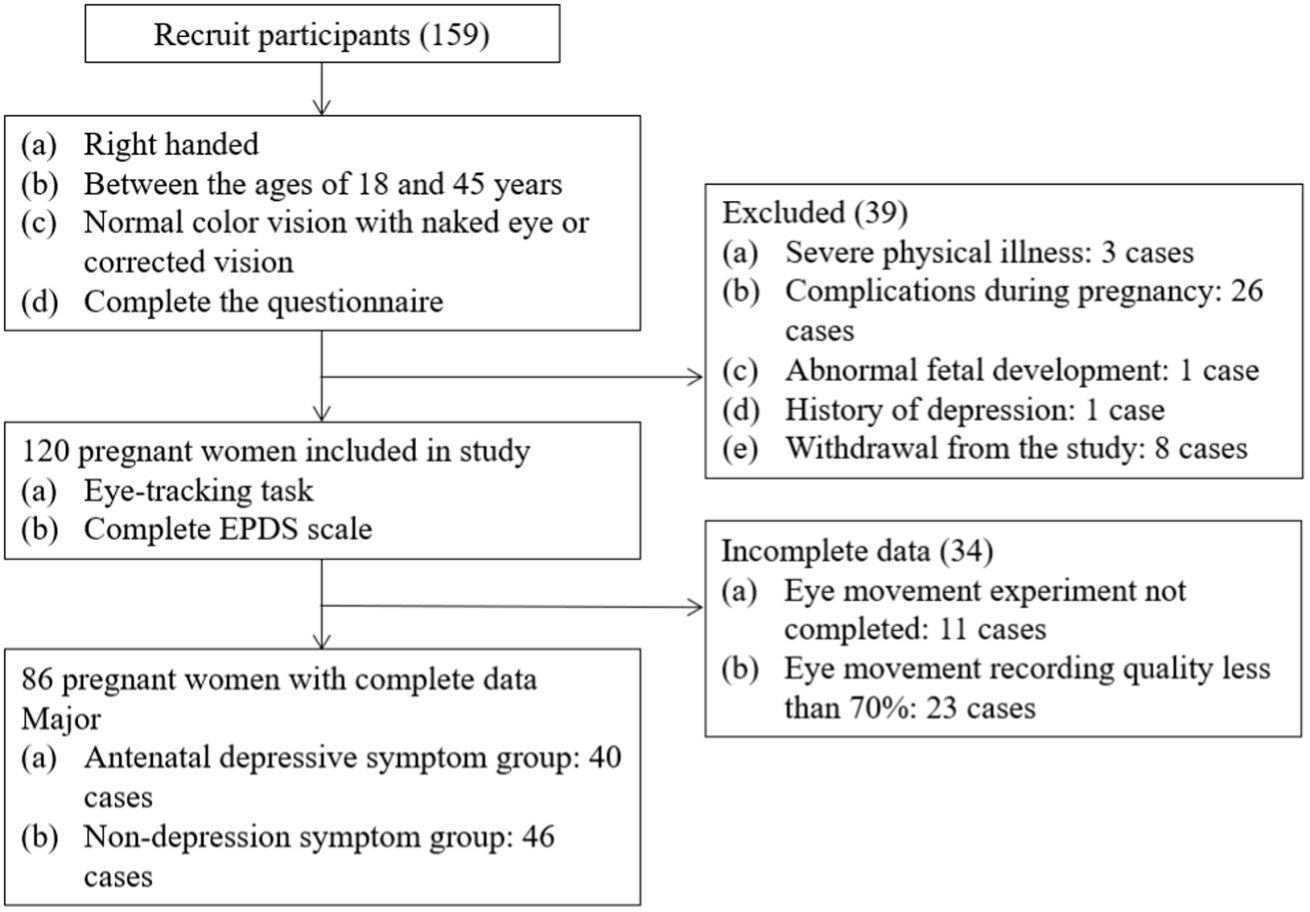
Study recruitment profile.

Figure 1 presents a flow chart of the study’s recruitment and grouping. Once written informed consent was obtained, the participants were required to complete the questionnaire, which included the Chinese version of the EPDS (Cox et al., 1987) as well as demographic and obstetric questions (such as gestational week, parity, number of abortions, and gestational complications). A psychiatric interview was then conducted by a trained psychiatric postgraduate to confirm the mental health status of each participant. Finally, 46 participants were included in the antenatal depressive symptom group (AD) and 40 participants in the non-depression symptom group (ND) according to the completion of eye movement.

### 2.2 Self-report measures

#### 2.2.1 General questionnaire

A general questionnaire was used to collect social demographic information, obstetric information, and other similar details from the participants. The following data were collected: name, age, years of education, place of residence, marital status, work pressure, family income level, previous history of mental illness, gestational week, due date, complications during pregnancy, and childbirth circumstances.

#### 2.2.2 Edinburgh postnatal depression scale

The Edinburgh Postnatal Depression Scale (EPDS) is the most widely used self-report screening tool for perinatal depression (Gibson et al., 2009; Kozinszky and Dudas, 2015; Thombs et al., 2015). Generally, scores above 9 indicate possible depression, while those above 13 indicate probable depression. The Chinese version of the EPDS has good reliability and validity. Research recommends a cut-off score of 8/9 for the Chinese screening tool (Zhao et al., 2015). In the current study, the EPDS was used as an initial screening measure with a cut-off score of 9. Pregnant participants that scored above 9 were considered to be depressed and so were assigned to the depressive symptom group. Those scoring 9 or below were considered to be non-depressed and were assigned to the control group (Usuda et al., 2017).

### 2.3 Eye-tracking paradigm

#### 2.3.1 Materials

Past studies have typically used emotional stimuli (words, images, and faces) to demonstrate the attentional bias in depression and infant faces to demonstrate maternal issues. In this study, emotional infant-related images were employed to evaluate the attentional bias of pregnant women with depressive symptoms. During the assessments, a total of 12 pairs of images were used. Each pair consisted of an infant-related emotional image or character, such as a crying baby, and a neutral image or character, such as a house. The infant-related emotional images varied in their emotional valence, being either neutral, negative, or positive, while the neutral images remained consistently neutral throughout the pairs. All images were selected from the International Affective Picture System (IAPS) (Lang and Bradley, 2007) and were rated by 50 college students for valence and arousal on a scale from 1 to 9 in a preliminary study. The mean valence scores (with standard deviations (SDs) in parentheses) of the positive, negative, and neutral infant-related images and the neutral images were 7.13, 2.92, 5.46, and 5.30, respectively. The mean arousal scores were 6.79, 6.97, 5.37, and 4.78, respectively. Finally, the mean infant relevance scores were 8.58, 7.40, 7.83, and 1.62, respectively. All stimuli were presented as 23-inch broadband images with a resolution of 1,024 × 768 pixels.

#### 2.3.2 Apparatus

The apparatus used in this study was a Tobii TX300 non-invasive infrared eye-tracker system. The eye-tracker denotes visual fixation as the period in which the participant focused his or her gaze within the area of interest (AOI). In this study, the AOI was the main content of each image. Fixation data were recorded with the eye-tracker for each AOI and were used to estimate sustained visual processing indices. The criteria for identifying an initial shift in gaze on each trial included (Caseras et al., 2007): (a) the participant was fixated in the central region before picture onset; (b) eye movements occurred after at least 100 ms and with a maximum fixation radius of 1° after picture onset and before picture offset (Lang and Bradley, 2007), and (c) gaze was directed to the picture (left or right) rather than remaining at the central position during picture presentation. The sampling rate of the system was 120 Hz, the evaluation accuracy was 0.5 level, and the distance between the eyes and the screen was 65 cm, which could accommodate 37 cm x 17 cm of head motion in the plane.

#### 2.3.3 Procedure

**Figure 2:**
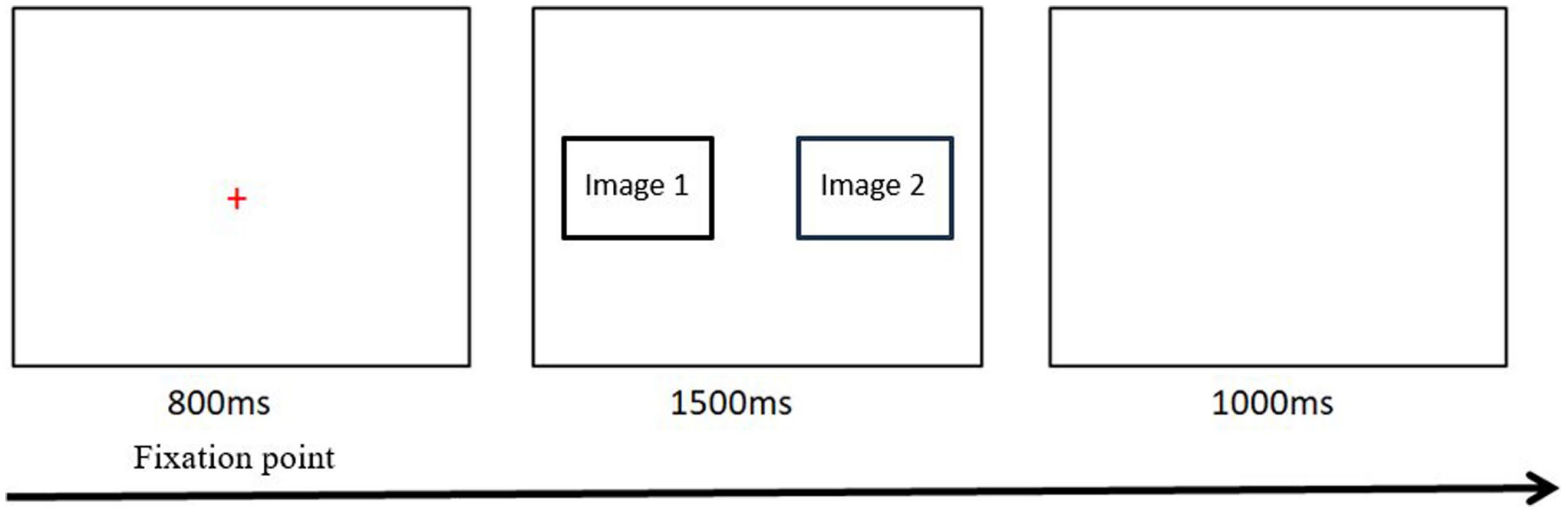
One trial flow diagram of free viewing task.

During the free-viewing task, the participants were seated on a sturdy chair in a comfortable position and viewed the screen from a distance of approximately 65 cm. To ensure optimum gaze data quality, the eye-tracker was calibrated for each individual (using a standardized five-point calibration procedure) before each attention task commenced. Emotional and neutral images were presented an equal number of times on the left and right sides of the screen. When displayed on the screen, each picture measured 600 × 400 mm, with the centers of each picture 600 mm apart. The task consisted of eight practice trials, followed by a brief pause (3 s) and then 48 experimental trials (12 pairs of images were repeated 4 times). Each attention task trial consisted of a fixation cross (presented centrally for 800 ms), followed by a pair of images that were displayed for 1,500 ms, followed by blank masking which was displayed for 800 ms. Participants were asked to look at the picture on the screen as if they were watching a television program (free viewing) but were instructed to return their focus to the central fixation cross whenever it appeared. The 48 stimulus pairs were presented in a different randomized order for each participant. The whole experiment was carried out in a quiet clinic in the hospital, and each subject was assessed separately (see Figure 2).

#### 2.3.4 Attentional indices

Using gaze data collected by the eye-tracker system, three attentional indices (Mogg et al., 2003) were extracted for the present study: (a) direction of initial gaze (i.e., percentage of first fixations on each AOI); (b) first-fixation latency (i.e., time elapsed until the first fixation occurs on each type of picture in each trial); and (c) first-fixation duration (i.e., duration of the first fixation made on each type of picture in each trial).

In accordance with the guidelines, we calculated the relative bias scores for each attention index associated with different emotion categories. To determine the initial gaze direction, we examined the percentage of trials in which participants first fixed their gaze on the infant-related emotional stimuli rather than the neutral picture. If the bias score is above 50%, it indicates a preference for looking at the infant-related emotional stimuli. Conversely, a score below 50% suggests a bias toward the neutral picture. For the first-fixation latency and first-fixation duration, we computed the bias scores by subtracting the corresponding values obtained for the neutral picture from those obtained for the infant-related emotional stimuli, as done in previous studies (Duque et al., 2014). Bias scores with first-fixation durations longer than zero were considered to be biased toward infant-related emotional stimuli, while bias scores with shorter durations than zero suggested a preference for neutral stimuli. First-fixation latency bias scores were the reverse (Duque et al., 2014; Veerapa et al., 2023).

On the basis of previous studies, the initial orientation bias score was used as the core index of H1 in this study, and the first fixation latency bias score and the first fixation duration bias score were used as auxiliary indexes (Soltani et al., 2020). The first fixation latency bias score was used as the core indicator of H2, and the initial orientation bias score and the first fixation latency bias score were used as auxiliary indicators (Veerapa et al., 2023).

### 2.4 Statistical analysis

Eye movement data were collected using Tobii Studio Software (2.0.6). Records of eye movement data quality is greater than 70% of the subjects were selected for data analysis. SPSS 24.0 was used for data analysis in this study. First, the demographic and clinical characteristics of the two groups of patients were compared using independent samples t-test, chi-square test, and Fisher’s exact test. Secondly, a 2 (group) × 3 (stimulus type) mixed design analysis of variance was conducted to examine the differences in initial orientation bias scores, first-fixation latency bias scores, and first-fixation duration bias scores in order to investigate the effects of group and infant-related emotional stimuli on attention bias (Soltani et al., 2020). Finally, one-sample *t*-tests were performed on the three bias scores to explore the attention bias patterns in the two groups. The initial orienting bias score was compared to a 50% level, while the first-fixation latency bias score and first-fixation duration bias score were compared to a 0 level (Duque et al., 2014; Veerapa et al., 2023).

## 3 Results

### 3.1 Analysis of group characteristics

The characteristics of each group were then analyzed. As shown in Table 1, there were no significant differences in the demographic characteristics between the two groups.

**Table 1:** Demographic and clinical characteristics.

	AD (*N* = 40)	ND (*N* = 46)	
	M (SD)	M (SD)	*p*
Age, years	29.28 (4.21)	28.24 (3.71)	0.23
Gestational week, weeks	33.85 (7.49)	33.10 (7.42)	0.64
Pre-pregnancy BMI, kg/m^2^	21.25 (3.50)	21.45 (2.90)	0.77
Current BMI, kg/m^2^	25.56 (3.70)	25.58 (2.74)	0.98
Education year, years	13.05 (2.26)	13.33 (2.30)	0.57
EPDS score	12.78 (2.35)	6.20 (1.77)	<0.001
	*n* (%)	*n* (%)	-
Married	40 (100%)	46 (100%)	-
Parity	-	-	-
No previous children	17 (42.5%)	21 (45.7%)	0.77
At least one child	23 (57.5%)	25 (54.3%)	-
Residence	-	-	-
Urban	18 (45.0%)	15 (32.6%)	0.27
Village	22 (55.0%)	31 (67.4%)	-
Pressure of work	-	-	-
Mild	27 (67.5%)	37 (80.4%)	0.19
Moderate	11 (27.5%)	9 (19.6%)	
Severe	2 (5.0%)	0	-
Household income	-	-	-
3,001–5,000	9 (22.5%)	7 (15.2%)	0.15
5,001–10,000	15 (37.5%)	27 (58.7%)	-
Over 10,000	16 (40.0%)	12 (26.1%)	-

AD, Antenatal depressive symptom group; ND, Non-depression symptom group; BMI, body mass index; EPDS, Edinburgh Postnatal Depression Scale. *p*-value presents difference to control. Independent *t*-test, chi-square test, or Fisher’s exact test.

### 3.2 Direction of initial gaze

A 2 (antenatal depressive symptom group, non-depression symptom group) × 3 (stimulus type: positive, negative, and neutral infant-related stimuli) mixed-design ANOVA was used to investigate the attention bias of the participants to the infant-related emotion images. The main effect of stimulus type was not significant, (*F* (2, 168) = 2.669, *p* = 0.072, *η*^2^ = 0.031) and the group × stimulus type interaction was not significant, (*F* (2, 168) = 0.175, *p* = 0.840, *η*^2^ = 0.002). The main effect of group was not significant, *F* (1, 84) = 3.553, *p* = 0.063, *η*^2^ = 0.41, with an average difference of 4.53 (95% confidence interval: −0.249 to 9.307). This indicates that there were no significant differences in the initial attentional orientation to infant-related images among the participants.

Comparison of each bias score with a value of 50% (no bias) indicated that the bias scores of the two groups of participants on the infant-related positive and neutral images were more than 50% (see Table 2).

**Table 2:** One-samples *T* tests for three bias indicators.

Bias scores		Direction of initial gaze (%)		First fixation mean latency (ms)		First fixation duration (ms)	
		AD	ND	AD	ND	AD	ND
IN-N	M	55.63	51.09	−88.28	−30.85	49.14	70.45
	SD	19.19	16.63	144.28	137.68	204.11	195.61
	*t*	1.85	0.44	−3.87	−1.52	1.52	2.44
	*p*	0.071	0.660	<0.001	0.136	0.136	0.019*
IP-N	M	53.03	52.90	−137.43	−96.24	133.76	63.86
	SD	10.56	9.69	144.21	134.86	184.48	194.47
	*t*	1.81	2.03	−6.03	−4.84	4.59	2.23
	*p*	0.078	0.049*	<0.001	<0.001	<0.001	0.031*
INeu-N	M	61.25	55.43	−147.04	−75.80	44.48	38.95
	SD	15.96	16.59	164.49	177.19	163.22	180.04
	*t*	4.46	2.22	−5.65	−2.90	1.72	1.47
	*p*	<0.001	0.031*	<0.001	0.006**	0.09	0.15

IN, infant-related negative image; IP, infant-related positive image; INeu, infant-related neutral image; N, neutral image; *t*, statistical value of one-sample *t*-tests; The initial orientation bias score test value was 50%, the first fixation mean latency bias score test value was 0, and the first fixation duration bias score test value was 0. *p < 0.05, **p < 0.01.

### 3.3 Bias score of the first fixation mean latency

A 2 (antenatal depressive symptom group, non-depression symptom group) × 3 (stimulus type: positive, negative, and neutral infant-related images) mixed-design ANOVA was used to investigate the bias of the latency scores to the infant-related emotional images. The results revealed that the group × stimulus type interaction was not significant, *F* (2, 168) = 0.275, *p* = 0.76, *η*^2^ = 0.003, but the main effect of group was significant, *F* (1, 84) = 6.175, *p* = 0.015, *η*^2^ = 0.068, with an average difference of −56.62 (95% confidence interval: −101.93 to −11.30). Compared with the neutral stimulus, depressed participants fixated on infant-related images faster than non-depressed participants. The main effect of stimulus type was also significant, *F* (2, 168) = 4.850, *p* = 0.009, *η*^2^ = 0.055. *Post hoc* LSD analyses found significant differences between infant-related negative and infant-related positive images (*p* = 0.007), with an average difference of 57.271 (95% confidence interval: 16.267 to 98.274); the latency bias score for the infant-related negative images was greater than that for the infant-related positive images, indicating that participants directed their first fixation to the positive images earlier.

Comparisons with a no-bias criterion (zero) indicated that the antenatal depressive symptom group tended to direct their first gaze to the infant-related images earlier (Table 2) and that the infant-related images were detected significantly faster than the neutral images. There was no significant bias in first gaze latency between infant-related negative and neutral images in the non-depression group.

### 3.4 Bias score of first fixation duration

The results of the mixed-design ANOVA indicated that the main effect of stimulus type was not significant, *F* (2, 168) = 2.251, *p* = 0.108, *η*^2^ = 0.026, and there was no significant interaction between group and stimulus type, *F* (2, 168) = 1.452, *p* = 0.237, *η*^2^ = 0.017. The main effect of group was also not significant, *F* (1, 84) = 0.511, *p* = 0.477, *η*^2^ = 0.006.

Comparisons with a no-bias criterion (zero) indicated that both groups of subjects had longer first fixations for infant-related positive emotions rather than neutral images. However, compared with neutral images, the non-depression group maintained a significantly longer fixation duration on negative images (see Table 2).

## 4 Discussion

This study used eye-tracking technology to assess the attention bias toward infant stimuli in pregnant women with depressive symptoms and a control group without depressive symptoms. In the following section, we will review the main findings of our study and their significance for understanding attention bias in pregnant women with antenatal depressive symptoms.

Previous studies have shown that increased attention to infant distressed during pregnancy is associated with a better connection between the woman and her child after childbirth, but women with perinatal depression have been found to pay significantly less attention to infant distressed; this suggests that healthy mothers pay more attention to the negative emotions of their infants than mothers with perinatal depression (Pearson et al., 2011; Rutherford et al., 2016). However, most published studies have used a response time-based paradigm represented by point detection methodology; such an approach cannot measure and distinguish specific attentional components (Waechter et al., 2014). The current study, for the first time, adopted a free viewing paradigm and eye-tracking technology to explore the characteristics and differences in the attention bias of pregnant women with and without antenatal depression to infant-related emotional images during 1,500 ms stimulations. The study findings provide a reference for further exploring the underlying mechanism of the attentional bias pattern in women with antenatal depression. The attentional bias indices for each type of infant-related emotional stimulus were calculated, including the initial fixation direction, the average latency of the first fixation, and the first fixation duration. The data demonstrated an attentional bias toward infant-related emotional images in pregnant women during the initial orienting and vigilance of attention, which is similar to previous studies (Lucion et al., 2017; Li et al., 2019). Individuals with depression have been reported to have an attentional bias to stimuli related to emotional disorders (Peckham et al., 2010). In the current study, compared to healthy pregnant women, pregnant women with depression symptoms more quickly directed their attention to infant-related images initially. This finding suggests that the orienting mechanism was not only selectively biased toward emotional stimuli (indexed by the probability of the first fixation) but also triggered faster by these stimuli (indexed by shorter saccade latencies) (Calvo et al., 2007). This conclusion is consistent with a previous study by our group (Tang et al., 2019). However, the emotional images utilized in the above study by Tang et al. were completely unrelated to infants. In the current study, the emotional stimuli were infant-related, which is a notable refinement of the previous experiment. Thus, this finding is of great significance for further understanding the mechanism of attentional bias in pregnant women with antenatal depression.

The second important finding of this study was that, relative to neutral stimulus, pregnant women focus on infant-related positive stimulus faster, and the first fixation mean latency bias score is shorter, possibly because images of positive emotions in infant stimulate maternal neural circuits to a greater extent. Studies have reported that there are regions of the brain that regulate “maternal” behavior, trigger and regulate the emotional responses, decision-making, and other parenting behaviors of mothers directed to their infants. The principal brain areas include the hypothalamic midbrain limbic cortical neural loop (Kim et al., 2010). Hoekzema et al. (2017) measured the volume of gray matter primiparas before pregnancy, early in postpartum, and again 2 years after delivery. It is proposed that this change in brain structure will affect a pregnant woman’s response to various aspects of fetal information (Kim et al., 2010).

In addition, the study found that the non-depression group had a longer first gaze duration for the negative images compared to the neutral images, while the antenatal depressive symptom group did not have this difference, which is consistent with previous results (Pearson et al., 2010). Previous studies have indicated that when babies are unhappy, it is difficult for pregnant women to look away from them (Pearson et al., 2011). This focus on baby distress may help women take better care of their children because crying and sadness are the ways babies send signals that they need help and attention (Pearson et al., 2011). Attention shifting is strongly associated with depressive symptoms (Liu et al., 2022), and this change in attentional bias pattern may be one of the reasons for the lack of maternal behavior in women with antenatal depression. On the other hand, functional magnetic resonance imaging (fMRI) evidence indicates that women with antenatal depression have abnormal brain function, including increased brain activity (low-frequency fluctuations in fractional amplitude) in areas of the left medial prefrontal cortex (MPFC), dorsalateral prefrontal cortex (DLPFC), and anterior cingulate cortex (ACC). Previous studies have shown that the DLPFC plays a key role in ‘top-down’ cognitive control, and the ACC is mainly involved in affective motivation and cognitive-attention (Ochsner and Gross, 2005; Ochsner et al., 2009; Wang et al., 2019). Thus, we speculate that the change in the attentional bias pattern may be related to changes in brain structure and function in women with antenatal depressive symptoms.

There are several limitations of the current study that need to be noted. Firstly, the measurement of depressive symptoms was principally based on the EPSD, so we were only able to identify women with depressive symptoms. Future research may use other objective scales to obtain clinical diagnoses. Secondly, only the cross-sectional effect of antenatal depressive symptoms on attentional bias to infant-related images was considered. The dynamic relationship between depressive symptoms and attentional bias patterns is not known. Further follow-up research is critical. Thirdly, there was no comparison group comprising non-pregnant women. Future studies should include non-pregnant women as an additional control group so that comparisons can determine the association between sensitivity to emotional stimuli and eye movement indicators. In addition, future research can combine magnetic resonance imaging technology to further investigate the correlation between prenatal depression and infant-related stimuli as well as attention bias. Finally, eye-tracking studies tend to have small sample sizes. Although the sample size in the current study was slightly larger than that of most eye movement studies, future studies should explore these preliminary findings with larger sample groups.

## 5 Conclusion

In conclusion, this study used a free viewing paradigm and eye-tracking technology to advance our understanding of attentional bias in antenatal women with depressive symptoms. The results showed that pregnant women with antenatal depressive symptoms had a faster first detection speed for infant-related images and had a significant attentional bias toward positive infant-related stimuli. We speculate that structural and functional changes in affective motivation and cognitive-attention brain areas may induce these changes in the attentional bias pattern. These results provide suggestions for future research and evidence to support the implementation of clinical intervention programs to correct the attention bias of depressed antenatal women.

## Data availability statement

The data presented in the study are deposited in the Figshare repository. This data can be found here: https://figshare.com/articles/dataset/__xlsx/24548398.

## Ethics statement

The study protocol was reviewed and approved by the Research Ethics Committee, Wenzhou Medical University, reference number 2018-KY043 before the research was carried out. The studies were conducted in accordance with the local legislation and institutional requirements. The participants provided their written informed consent to participate in this study.

## Author contributions

YX: Data curation, Methodology, Writing – original draft, Writing – review & editing. PZ: Writing – original draft. WF: Methodology, Writing – original draft. LC: Writing – original draft, Writing – review & editing. SS: Writing – original draft. JL: Writing – original draft. WT: Conceptualization, Methodology, Supervision, Writing – original draft. CB: Writing – original draft. LX: Writing – original draft. DX: Writing – original draft, Writing – review & editing. KZ: Project administration, Resources, Writing – original draft, Writing – review & editing.
